# Anesthetic Management for Headscarf Pin Tracheobronchial Foreign Body Removal in a Pediatric Patient

**DOI:** 10.1155/carm/4664553

**Published:** 2026-04-09

**Authors:** Amadou Diaw Diop, Faboye Ndoumbe Mathilde Diop, Alpha Diallo, Lijun Chen, Wenqing Zhang

**Affiliations:** ^1^ Department of Anesthesiology, Children’s Hospital of Diamniadio, Dakar, Senegal; ^2^ Zhangzhou Hospital of Traditional Chinese Medicine, Zhangzhou, Fujian, China; ^3^ Department of Anaesthesiology, Zhangzhou Affiliated Hospital of Fujian Medical University, Zhangzhou, Fujian, China, zzfh.com

**Keywords:** airway obstruction, bronchial foreign body, case report, deep sedation, foreign body removal, rigid bronchoscopy, spontaneous respiration

## Abstract

Foreign bodies in the airways are relatively common in preschool‐ and school‐aged children. We report a rare case of the successful removal of a sharp metallic foreign body from the bronchus of a 12‐year‐old boy under combined inhalational and intravenous anesthesia without tracheal intubation. This report emphasizes the perioperative anesthetic strategies for foreign object removal. The patient presented with sudden‐onset choking and hemoptysis. Chest computed tomography revealed a linear metallic foreign body measuring approximately 5 cm long in the left main bronchus. The foreign body was removed via rigid bronchoscopy under deep sedation maintained by a combination of inhalational and intravenous anesthesia while preserving spontaneous respiration. Intraoperative ventilation was controlled intermittently to ensure adequate oxygenation. Based on recent studies, we discuss key anesthetic considerations and risk‐mitigation strategies for managing sharp bronchial foreign bodies in pediatric patients. This case highlights the importance of maintaining spontaneous respiration during bronchoscopy and tailoring anesthetic approaches to ensure safe outcomes, despite limited access to advanced airway technologies.

## 1. Introduction

Tracheobronchial foreign bodies are common and potentially life‐threatening in pediatric patients. Although cases of foreign body obstruction involving sharp metallic objects, such as sewing or headscarf pins are rarely reported in the Chinese literature, they are more commonly documented in other countries, especially in Arab regions, where such pins are frequently used to secure headscarves or veils [1]. The average age of affected children is approximately 3 years, with 53%–65% being boys [2]. The sharp ends of these pins may penetrate the airway wall, causing mediastinal emphysema or major vascular injury. Anesthetic management in these cases presents unique challenges. The 2022 American Society of Anesthesiologists (ASA) Practice Guidelines for Management of the Difficult Airway [3] emphasize the need for individualized ventilation strategies and multidisciplinary contingency planning. Herein, we report a rare case of the successful removal of a sharp metallic bronchial foreign body under combined inhalational and intravenous anesthesia performed without tracheal intubation.

## 2. Case Presentation

### 2.1. Investigations

A 12‐year‐old boy (weight: 28 kg) from the suburbs of Dakar, Senegal, was admitted to the emergency department 2 h after accidentally swallowing a headscarf pin out of curiosity. Upon examination, his oxygen saturation (SpO_2_) was 96% at room air. There were no signs of cyanosis, stridor, or dyspnea. His history revealed a sudden, severe choking episode followed by persistent cough, odynophagia, reflex vomiting, facial flushing, and a sensation of breathlessness after aspiration, consistent with the so‐called “penetration syndrome” [4]. This syndrome represents the typical mucosal response when a sharp foreign body penetrates the larynx and enters the trachea. After lodging in the main bronchus, the foreign body entered a brief “quiet phase.” Persistent retention subsequently triggered early inflammatory changes in the left lung. Clinical manifestations, including low‐grade fever and worsening cough, indicated progression to the “irritation or inflammatory phase.”

Medical history: Preoperative inquiry with the patient’s family confirmed no history of respiratory diseases, such as asthma, pneumonia, or bronchitis, nor any bleeding tendencies or drug allergies. The patient’s psychosocial history was unremarkable. The patient was at an age characterized by strong curiosity and exploratory tendencies. He had no history of related behavioral or psychological abnormalities. Living in a suburban area with relatively rudimentary conditions, parental supervision was inconsistent, leading to occasional incidents of accidental ingestion or aspiration. This instance represents his first airway foreign body event, with no prior history of similar interventions. The patient had no history of hospitalization or treatment for other conditions (e.g., recurrent pneumonia).

Preoperative assessment: Postoperative assessment​ focused on pulmonary examination. Although visual inspection revealed no signs of respiratory distress, the respiratory rate was slightly elevated. No signs of upper airway obstruction, such as the “three retractions” (supraclavicular fossa, intercostal spaces, or subxiphoid retractions during inspiration), were observed.

Auscultation: After entering the operating room, bilateral lung sounds were auscultated, revealing unilateral (left‐sided) decreased breath sounds. The mechanical irritation and obstruction of the airway by the foreign body persisted; however, no signs of obstruction, such as cyanosis, wheezing, or dyspnea, were present.

Percussion: Percussion of the left chest revealed no dullness or hyperresonance, suggesting that the sharp foreign body had not yet caused atelectasis or obstructive emphysema.

**FIGURE 1:**
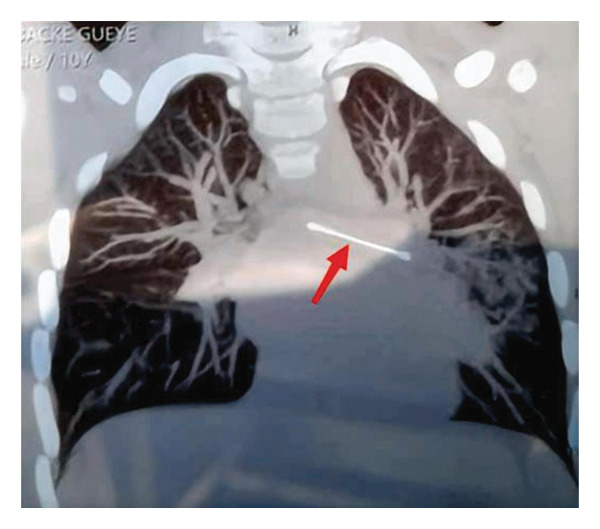
Chest computed tomography showing a striated hyperdense shadow in the left main bronchus (red arrow).

Imaging: Chest computed tomography (CT) showed no evidence of anatomical variations in the bronchial tree; however, it revealed a linear metallic foreign body shadow measuring approximately 5 cm in length within the left main bronchus, along with multiple patchy infiltrates in the left lung (Figure 1). The location of the foreign body and the patient’s detailed clinical condition were confirmed. The timeline of the patient’s illness onset and emergency department treatment process are detailed in Table 1.

**TABLE 1:** Timeline of the patient’s onset of illness and emergency department care.

Time of event	Action key/clinical manifestation	Finding/management	Decision
2 h before admission (T − 2 h)	Accidental swallowing of a headscarf pin	Sudden severe choking cough, swallowing pain (penetration syndrome)	Family rushed patient to hospital
Emergency admission (T0)	Initial medical contact	Complaint of swallowing pain, no cyanosis or wheezing; SpO_2_, 96%	Conducted initial assessment, prescribed urgent imaging
30 min after admission (T + 0.5 h)	Chest computed tomography (CT) scan	Noncontrast CT confirmed approximately 5 cm metallic foreign body shadow in the left main bronchus, patchy left lung infiltrates	Confirmed diagnosis; decision made for bronchoscopic foreign body removal
1 h after admission (T + 1 h)	Preoperative anesthesia assessment	Evaluated airway risk; confirmed no history of ingestion (managed as fasting patient)	Developed anesthesia plan (planned general anesthesia with spontaneous breathing or controlled ventilation strategy); prepared for surgery
(Planned) 2 h after admission (T + 2 h)	Bronchoscopic surgery planned	—	Planned foreign body removal via rigid or flexible bronchoscopy under general anesthesia in the operating room

### 2.2. Diagnosis

Noncontrast chest CT revealed a linear metallic foreign body measuring approximately 5 cm in length within the left main bronchus, accompanied by patchy infiltrates in the left lung (Figure 1). The diagnosis of a metallic foreign body in the left main bronchus in this case was primarily based on the following examinations.

Medical history: A clear history of foreign body aspiration (accidental ingestion of a hairpin) and the presence of a typical “penetration syndrome” (sudden, severe choking cough) provided the primary diagnostic clues.

Physical examination: Auscultation of both lungs revealed decreased breath sounds on the left side.

Chest CT (noncontrast): This was the definitive diagnostic test. For radiopaque foreign bodies such as the metal hair pin in this case, CT clearly showed the precise location (left main bronchus), size (5 cm), and shape (rod‐like). The CT findings of “multiple patchy infiltrates” in the left lung provided direct evidence of secondary infectious or obstructive pneumonia caused by the foreign body obstruction.

Therefore, the diagnosis was confirmed through a combination of a clear history of aspiration, typical clinical symptoms, and direct CT visualization of a metallic foreign body within the left main bronchus.

### 2.3. Treatment

#### 2.3.1. Preoperative Preparation

Airway evaluation*:* Preoperative chest CT showed a 5‐cm linear metallic object in the left main bronchus, with surrounding infiltrates in the left lung. The team conducted a preliminary assessment of the size, location, and depth of the foreign body impaction. However, potential airway injuries (e.g., perforation, bleeding, and edema) or secondary pulmonary infections could not be definitively excluded. The patient was also evaluated for complex airway risk factors; mouth opening, thyromental distance, neck mobility, and the Mallampati score were all within normal limits.

**FIGURE 2:**
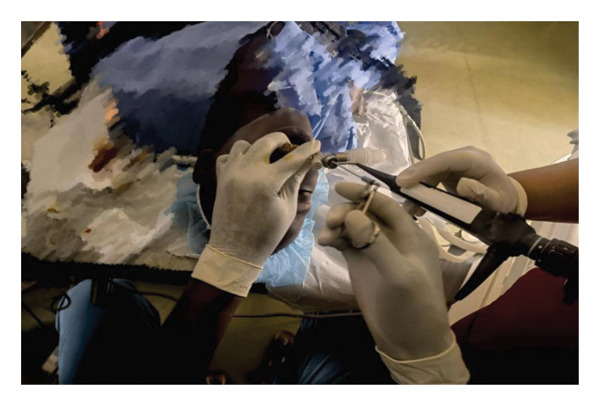
Soft fiberscope‐assisted rigid bronchoscopy to guide clamping of the foreign body during airway assessment.

Multidisciplinary collaboration*:* A multidisciplinary team of anesthesiology, otolaryngology/thoracic surgery, and critical care developed a surgical and anesthetic plan, along with contingency protocols. The sharp metallic foreign body in the bronchus was removed using rigid bronchoscopy. Given the restricted working space caused by the elongated foreign body lodged in the bronchus and a narrowed bronchial lumen, a video laryngoscope was used to assist the insertion of the rigid bronchoscope into the trachea. To determine the sharp object’s position and extent of embedding in the tissue, a flexible bronchoscope was inserted through the rigid bronchoscope’s working channel. The surgical team evaluated the removal direction and force, while monitoring for perforation, massive bleeding, and pneumothorax with this maneuver (Figure 2).

Patient preparation*:* Preoperative fasting was initiated per the elective surgery protocol since the patient showed no respiratory distress: 8 h for solids and 2 h for clear fluids. Upon entering the operating room, high‐flow oxygen was administered via face mask (FiO_2_ 1.0) for 5 min. Intravenous access was established, and lactated Ringer’s solution was infused. To prevent airway edema, dexamethasone (0.1 mg/kg IV) was administered. The ASA practice guidelines for challenging airway management require individualized ventilation and emergency preparedness [3]. Therefore, emergency airway equipment was readily available, including a video laryngoscope, fiberoptic bronchoscope, laryngeal mask airway (LMA), cricothyrotomy kit, and high‐frequency jet ventilation (HFJV). Emergency tracheostomy and extracorporeal membrane oxygenation equipment were also arranged.

### 2.4. Anesthesia Induction and Intraoperative Management

**FIGURE 3:**
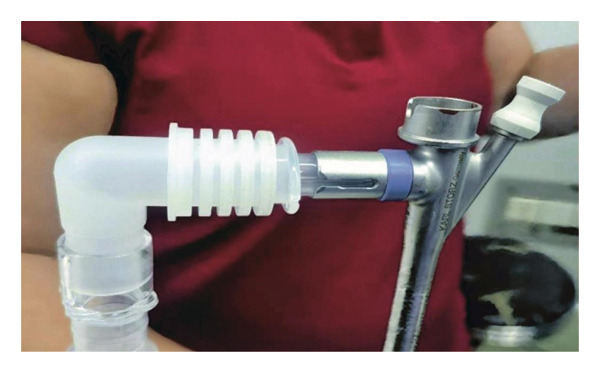
A 7.0# tracheal tube connector with a rigid bronchoscope side hole connection.

**FIGURE 4:**
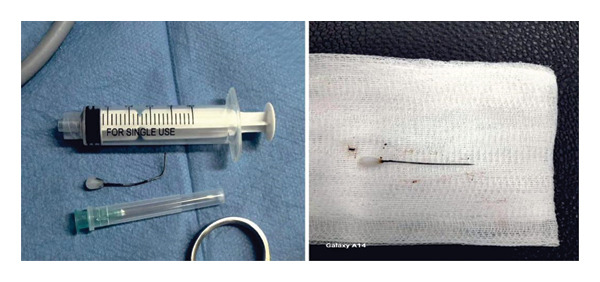
Sharp foreign body removed (turban needle, approximately 5 cm long).

A French study [5] on the anesthetic management of pediatric tracheobronchial foreign bodies recommended spontaneous respiration and avoiding positive pressure ventilation to reduce foreign body displacement during airway manipulation. Other studies suggest that positive‐pressure ventilation or HFJV may be safe when a foreign body is in the bronchus but has not yet caused airway obstruction [6]. In this case, after the child was brought to the operating room, propofol 2 mg/kg was administered intravenously for sedation, followed by 2% sevoflurane to preserve spontaneous respiration. A rigid bronchoscope was implanted, and propofol 2 mg/kg bolus was administered to deepen the anesthesia. When needed, the rigid bronchoscope’s side port was connected to the distal connector of a 7.0‐mm endotracheal tube for controlled ventilation (Figure 3). During the procedure, a sharp metallic foreign body was found embedded, with the tail end posing a risk of penetrating the airway wall. The forceps position had to be adjusted multiple times for safe extraction, prolonging the procedure. Maintaining deep anesthesia required repeated propofol bolus injections. To sustain oxygenation, aided and short‐term regulated breathing were used during respiratory depression. After the foreign body was successfully removed (Figure 4), an LMA was positioned to continue pressure‐controlled ventilation. Following the successful extraction of the foreign body, immediate intraoperative assessment, including visual confirmation of lung re‐expansion and bilateral equal breath sounds on auscultation, indicated restored airway patency, which is a key indicator for avoiding an immediate repeat CT scan. Nebulized epinephrine (3 mL of a 1:1000 solution) was administered to prevent postoperative laryngeal edema. The patient was extubated and transferred to the pediatric intensive care unit for 24‐h observation.

### 2.5. Follow‐Up and Outcomes

#### 2.5.1. Short‐Term Postoperative Recovery

Vital signs and oxygenation: Closely monitored within 24 h postoperatively, the patient’s heart rate, blood pressure, respiratory rate, and SpO_2_ were recorded and remained stable. SpO_2_ was maintained above 95% (without supplemental oxygen or low‐flow oxygen), indicating patent airways and adequate oxygenation.

Respiratory assessment: Recovery was evaluated through daily lung auscultation. Left‐sided breath sounds gradually became clear, and wheezing resolved. Concurrently, irritative coughing significantly diminished and eventually ceased.

Imaging follow‐up: A chest CT was repeated within 24 h postoperatively. It confirmed complete removal of the foreign body, demonstrated the beginning of absorption of patchy infiltrates in the left lung, and ruled out early complications such as pneumothorax or atelectasis.

### 2.6. Symptom Relief and Functional Recovery

The patient’s primary preoperative symptoms—dysphagia and irritative cough—rapidly resolved after foreign body removal. By postoperative Day 1, coughing had markedly decreased, and the patient tolerated a liquid diet without difficulty. Anesthetic recovery was uneventful with no agitation, and spontaneous breathing resumed effectively, reflecting the appropriateness of the anesthetic protocol.

## 3. Discussion

### 3.1. Choice of Ventilation Strategy

Recent studies have confirmed that HFJV can significantly reduce the risk of barotrauma compared with conventional ventilation during airway foreign body removal procedures [7]. However, in resource‐limited settings where advanced equipment may not be available, conventional methods must be adopted. In this case, we preserved the patient’s spontaneous respiration and devised a simple yet effective ventilation approach in collaboration with the otolaryngology team. A custom‐made adapter was used to connect the side port of the rigid bronchoscope to a standard endotracheal tube connector. This action allowed for a hybrid ventilation strategy combining spontaneous breathing with intermittent assisted ventilation and high‐frequency PCV. This approach prevented foreign body displacement while maintaining adequate oxygenation throughout the procedure, and the patient did not experience any episodes of hypoxemia.

### 3.2. Strengths and Limitations

The most challenging aspect of maintaining spontaneous breathing during endotracheal foreign body removal is controlling the depth of anesthesia. If the anesthesia is too deep, maintaining spontaneous breathing becomes difficult. Therefore, it is essential to monitor spontaneous breathing during the procedure and perform assisted breathing or high‐frequency ventilation to maintain the oxygen supply if necessary. If the anesthesia is too shallow to maintain spontaneous breathing, intraoperative tracheoscopic manipulation may trigger the patient’s involuntary motor response or even lead to serious complications such as displacement of the sharp foreign body or hemorrhage of important blood vessels. Therefore, there are advantages and disadvantages of maintaining spontaneous breathing during the operation, and close monitoring must be performed on the premise of strictly guaranteeing the depth of anesthesia.

### 3.3. Controversy Regarding the Use of Muscle Relaxants

While some studies support the use of neuromuscular blocking agents and inhalational anesthesia to provide a consistent and adequate depth of anesthesia during rigid bronchoscopy [8], this approach remains controversial in cases involving sharp tracheobronchial foreign bodies. The sharp end of the object may become deeply embedded in the airway wall during muscle paralysis, thereby increasing the risk of iatrogenic injuries. In this case, we avoided muscle relaxants and opted for deep sedation while preserving spontaneous respiration, as a multimodal anesthesia regimen combining sevoflurane and propofol (other options may include ketamine, which was not used in this case) achieved a satisfactory depth of sedation and airway control. A multimodal anesthesia regimen combining sevoflurane, propofol, and ketamine can be used to achieve a satisfactory depth of sedation and airway control. The rigid bronchoscope was introduced into the trachea under video laryngoscopic guidance. Subsequently, the flexible fiberscope was advanced through the working channel of the rigid bronchoscope. This facilitated direct visualization and assessment of the foreign body’s position and embedding before any extraction attempt with forceps. The optical system of the rigid bronchoscope itself was also used for general navigation.

### 3.4. Value of Imaging‐Guided Navigation

Preoperative virtual bronchoscopy based on three‐dimensional CT reconstruction can provide precise localization of the foreign body’s orientation, shape, and degree of tissue embedding. This information is invaluable for both the surgical and anesthesia teams when planning the procedure and anticipating potential complications. However, intraoperative events remain unpredictable, and the removal of sharp foreign bodies is often technically more challenging than expected. In this case, the foreign body became repeatedly lodged in the bronchial wall, prolonging surgery and making the management of anesthetic depth more challenging. A video laryngoscope was used to guide the rigid bronchoscope into the trachea to minimize injury during insertion.

## 4. Conclusion

The anesthetic management of sharp tracheobronchial foreign bodies in children should adhere to the principle of “minimal interference,” with emphasis on preserving spontaneous respiration whenever feasible. A targeted approach involving multimodal sedation, topical airway anesthesia, and the judicious use of assisted or controlled ventilation can help minimize the risk of foreign body displacement and airway trauma. Multidisciplinary collaboration, along with imaging‐guided navigation, plays a critical role in enhancing procedural safety and optimizing clinical outcomes. This case highlights the importance of individualized anesthetic planning and adaptability, particularly in resource‐constrained environments, for the successful removal of sharp airway foreign bodies in pediatric patients.

## Author Contributions

Conceptualization, Wenqing Zhang and Lijun Chen; methodology, validation, formal analysis, writing–review and editing, and visualization, Amadou Diaw Diop and Wenqing Zhang; software, Alpha Diallo and Amadou Diaw Diop; investigation, Lijun Chen and Faboye Ndoumbe Mathilde Diop; resources, Amadou Diaw Diop, Faboye Ndoumbe Mathilde Diop, and Wenqing Zhang; data curation, Alpha Diallo and Lijun Chen; writing–original draft preparation, Alpha Diallo, Faboye Ndoumbe Mathilde Diop, and Alpha Diallo; supervision, Wenqing Zhang; project administration, Faboye Ndoumbe Mathilde Diop and Wenqing Zhang.

## Funding

The authors received no financial support for the research authorship and/or publication of this article.

## Disclosure

All authors have read and agreed to the published version of the manuscript.

## Ethics Statement

This study was approved by the Comite’ d’Ethique Medicale of the Université Iba Der THIAM de Thiès, following Senegalese law (Reference no. CEM/[2025]/[10625]) and was conducted in compliance with the Declaration of Helsinki (https://www.wma.net/policies-post/wma-declaration-of-helsinki/).

## Consent

This study was authorized by the patient’s legal guardians. No other authorization was required for the investigation. Written informed consent to publish was obtained from the patient’s legal guardians.

## Conflicts of Interest

The authors declare no conflicts of interest.

## Data Availability

All data generated or analyzed during this study are included in this published article.
